# Longitudinal effects of exercise according to the World Health Organization guidelines on cognitive function in middle-aged and older adults

**DOI:** 10.3389/fpubh.2022.1009775

**Published:** 2022-10-28

**Authors:** Dasom Kim, Young Ko, Aeri Jung

**Affiliations:** ^1^Expert Group on Health Promotion for Seoul Metropolitan Government, Home Visit Healthcare Team, Seoul, South Korea; ^2^College of Nursing, Gachon University, Incheon, South Korea; ^3^College of Nursing, Eulji University, Uijeongbu, South Korea

**Keywords:** aged, cognition, mental status and dementia tests, physical activity, longitudinal analysis

## Abstract

**Purpose:**

To investigate the longitudinal effects of adequate exercise, defined as an exercise duration of ≥150 min/week by the World Health Organization (WHO), on cognitive function in middle-aged adults.

**Methods:**

This study was a longitudinal panel analysis using secondary data obtained from the Korean Longitudinal Study of Aging (KLoSA) database, with 4,825 participants registered and comprising five rounds of survey data in 2-year intervals from 2010 to 2018. The participants were divided into the adequate exercise (≥150 min/week), deficient exercise (<150 min/week), and no exercise groups according to the WHO definition, and their cognitive decline over the 8-year period was analyzed. Further, we investigated the longitudinal effects of exercise using a fixed effects model with cognitive function as the dependent variable.

**Results:**

In the dementia group, both deficient (<150 min/week) and adequate (≥150 min/week) exercises had statistically significantly positive effects on cognitive function. However, the coefficient size was not significantly larger in the adequate exercise group than in the deficient exercise group. In the participants with mild cognitive impairment (MCI), an adequate exercise level had significantly positive effects on cognitive function, while a deficient exercise level did not. In the participants with normal cognition, an adequate exercise level was not significantly associated with changes in cognitive function.

**Conclusion:**

Continuous exercise can have a positive influence on cognitive function scores in middle-aged and older adults with MCI or dementia, but the findings cannot substantiate that adequate exercise (≥150 min/week) is more effective compared to deficient exercise (<150 min/week).

## Introduction

The prevalence of dementia among older adults aged ≥60 years is reported to be ~5% globally, but the absolute number of patients with dementia is anticipated to increase as a result of the growing elderly population. In particular, the entire baby boomer generation in Korea will enter the older adulthood stage by 2028, thereby increasing the elderly population to 17.22 million by 2040. In other words, the elderly population is estimated to double in size in ~20 years from 8.13 million in 2020. Furthermore, with a decreasing fertility rate, the old age dependency ratio is projected to triple from 22.4 in 2020 to 61.6 in 2040, highlighting a looming national elderly caregiving crisis along with escalating health care burden (1, 2).

The currently known non-modifiable dementia risk factors include gene polymorphisms, age, sex, race, and family history, and although age is a potent risk factor, dementia is not considered a normal aging process. Modifiable dementia risk factors include educational level, alcohol use, smoking, malnutrition, reduced physical activity, chronic diseases, such as hypertension, diabetes mellitus, and hypercholesterolemia, obesity, depression, social isolation, and poor cognitive activity (3–6).

Among these factors, physical activity and cognitive function—the focuses of this study—can potentially lower the risk for cardiovascular disease (CVD), hypertension, insulin resistance, and hypercholesterolemia indirectly. According to the frontal aging hypothesis, physical activity delays neuronal destruction by increasing the blood flow to the brain and level of brain-derived neurotrophic factor (BDNF) (7). Further, some studies suggest that physical activity does not influence the entire cognitive function but rather only the cognitive function related to the frontal lobe. The frontal lobe is responsible for the following cognitive functions: short-term memory, concentration, immediate memory, delayed memory, language proficiency, and frontal motor function (8, 9). In particular, studies showed that aerobic exercise is significantly effective by increasing the brain blood flow through the improvement of the cardiovascular functions (9).

Individuals who maintained physical activity in middle adulthood reportedly show a larger brain volume and considerably larger gray matter volume on magnetic resonance imaging (MRI) scans ~20 years later than those who maintained a sedentary lifestyle (10). This suggests that long-term physical activity can mechanically alter the brain structure and that even short-term exercise is linked to brain activity.

The 2019 WHO Guidelines for Risk Reduction of Cognitive Decline and Dementia recommend physical activity to maintain cognitive function in older adults. However, more specifically, the level of evidence for physical activity preventing cognitive decline in normal older adults was moderate, whereas that among older adults with a diminished cognitive function was low (11). Systematic reviews and meta-analyses reported that physical activity helps individuals maintain their ability to perform the activities of daily living (ADLs) and lowers the psychomotor symptoms of dementia and depression in older adults with mild cognitive impairment (MCI) and dementia; however, the direct effects of exercise on cognitive function remain controversial (12–15). Nevertheless, the WHO guidelines for physical activity in older adults recommend exercising for ≥150 min/week and stress the importance of muscle and aerobic exercises.

However, no study has conducted a longitudinal follow-up to examine the effects of long-term engagement in an adequate level of exercise (≥150 min/week according to the WHO recommendation) on cognitive function and to investigate whether an adequate exercise level serves as a cognitive protective factor by comparing individuals with an adequate exercise level with those without an adequate exercise level (<150 min/week). Research utilizing long-term data is considered as highly significant because the adequate exercise affects the cognitive function, and the effect accumulate over time.

In this context, this study aimed to longitudinally analyze the levels of exercise among middle-aged and older adults with normal cognition, MCI, or dementia in Korea from 2010 to 2018 and identify the effects of an adequate exercise level on cognitive function using a fixed effects model that utilizes the Korean Longitudinal Study of Aging (KLoSA) data.

The present study aimed to (1) longitudinally analyze the levels of exercise in middle-aged and older adults with normal cognition, MCI, or dementia using panel data; (2) examine the longitudinal effects of an adequate exercise level according to the WHO recommendation on cognitive function; and (3) examine the longitudinal effects of an adequate exercise level according to the WHO recommendation on the cognitive function of adults with normal cognition, MCI, and dementia.

## Methods

### Study design

The present study is a longitudinal panel analysis using secondary data (KLoSA) comprising five rounds of survey data collected in 2-year intervals from 2010 to 2018.

### Participants

We downloaded the light version of the KLoSA data and used the five datasets collected for the same parameters in 2-year intervals from 2010 (3rd survey) to 2018 (7th survey). The data used in this study were open access data that do not contain personal information; thus, the present investigation is not considered a human study. Accordingly, the study was exempted for review by the Institutional Review Board (IRB) at Gachon University (IRB No. 1044396-202202-HR-040-01). From the data of 5,672 participants matched from the 7th survey to the 3rd survey in reverse order based on the panel ID, the data of 4,825 participants who were determined to have normal cognition based on the Mini Mental State Examination (MMSE) in the 3rd survey (2010) were analyzed.

The KLoSA was first conducted in 2006 by sampling from home-dwelling individuals aged ≥45 years in regions in Korea, excluding Jeju Island. Beginning with the 1st survey in 2006, basic general surveys including the same parameters were repeated every other year, with the 7th survey completed in 2018. First, the study population was stratified by region and living arrangement, and 1,000 sample enumeration districts were extracted. A systematic sampling method was used to extract sample households per the designated sampling rate. The enumerators visited the sample households in order and determined the eligibility of each household, i.e., at least one member of the household was aged ≥45 years. All household members aged ≥45 years were interviewed.

### Instruments

#### Demographic characteristics

In this study, the factors reported to potentially influence cognitive function, namely age, educational level, sex, marital status, socioeconomic status (SES), depression, and health behaviors were selected as confounders (5, 6, 11, 16–19) to effectively infer the effects of exercise on cognitive changes.

The educational level was determined based on the highest completed educational level and was classified into elementary school, middle school, high school, and college or higher. A higher educational level was given a higher value. For marital status, 0 was coded for unmarried (separated, widowed, and divorced) and 1 was coded for married or living with a partner. SES was determined based on the personal allowance per month (unit: USD), which better reflects the excess resources available for use in daily life, as compared with net worth or debt.

Social contact frequency refers to the frequency of meeting a close social contact, such as friend, neighbor, or relative, in the past year.

#### Cognitive function

The dependent variable in this study is the presence of cognitive decline, and cognitive function was assessed using the Korean version of the MMSE (K-MMSE), which comprises 30 items for orientation, memory, attention and computation, language, and spatiotemporal aspect. The participants were classified as having suspected dementia (K-MMSE ≤ 17), cognitive impairment (K-MMSE 18–23), and normal cognitive function (K-MMSE ≥ 24) (4). Further, the participants with normal cognition, cognitive impairment, suspected dementia, and no response or with missing value were coded with 0, 1, 2, and 3, respectively.

#### Exercise sufficiency

Weekly exercise frequency and duration (minutes) in the 3rd−7th KLoSA database were used. The responses were interpreted for exercise sufficiency in older adults according to the WHO guidelines (11) to code the participants with 0 for no exercise, 1 for deficient exercise frequency or duration, and 2 for adequate exercise frequency or duration as defined as at least 30 min of per session for at least ≥5 days/week or at least 150 min/week.

### Analysis

First, the data were analyzed using STATA 17.0. The participants were divided into three groups (normal cognition, MCI, and dementia) based on the K-MMSE score in the 7th survey (2018). Missing values were replaced by generating five datasets using multiple imputations, and all variables used in the fixed effects analysis were considered in the multiple imputation model. Furthermore, five datasets were synthesized in the final fixed effect model for analysis (20).

Second, for the demographic characteristics, SES, depression, cognitive function, and health behaviors, categorical variables were expressed as frequencies and percentages, and continuous variables as means and standard deviations. To analyze the exercise status by cognitive function, the between-frequency and between-proportion data for the adequate exercise (≥150 min/week), deficient exercise (<150 min/week), and no exercise groups were analyzed. Moreover, the degree of cognitive decline over time was graphed for each group (adequate, deficient, and no exercise groups).

Third, we examined the longitudinal effects of adequate exercise on cognitive function by setting the cognitive function as the dependent variable and entering the demographic factors and other confounders into a fixed effects model using panel data. The fit of the fixed and random effects models were compared using the Hausman test. The fixed effects model was chosen, because the null hypothesis was rejected. A fixed effects model shows whether an dependent variable can be altered by changing the independent variable included in the model; if the independent variables do not have fixed effects on the dependent variable, it is highly likely to be due to inter-individual heterogeneity not observed in the study. Therefore, if an adequate exercise level has significant fixed effects on cognitive function in a model, we can infer that an increase in an individual's exercise level affects cognitive function over time even after controlling for the effect of innately lower cognitive function compared with other individuals (21).

Therefore, this enables a clearer inference of the causal effects of alterations of the independent variable on the dependent variable over time, in contrast to the regression analysis performed in cross-sectional studies.

## Results

### General characteristics of the participants

Table 1 shows the general characteristics of the participants stratified by their level of cognition (normal cognition, MCI, and dementia groups). The mean age was 60.14 ± 7.96, 65.61 ± 8.08, and 69.46 ± 9.06 years in the normal cognition, MCI, and dementia groups, respectively. Given that cognitive decline occurs in ages ≥65 years, the MCI and dementia groups were older than the normal cognition group in the 7th survey.

**Table 1:** Descriptive analysis of the characteristics of the participants in the 2010 survey (3rd wave).

**Variable**	**Categories**	**Normal (*N* = 3,341)**	**MCI (*N* = 694)**	**Dementia (*N* = 250)**
		**Mean** ±**SD or** ***N*** **(%)**		
Age		60.14 ± 7.96	65.61 ± 8.08	69.46 ± 9.06
Sex	Male	1,590 (47.59)	289 (41.64)	113 (45.20)
	Female	1,751 (52.41)	405 (58.36)	137 (54.80)
Education	Elementary school	956 (28.62)	405 (58.36)	154 (61.6)
	Middle school	679 (20.33)	124 (17.87)	44 (17.6)
	High school	1,260 (37.72)	138 (19.88)	38 (15.2)
	Above college	445 (13.32)	27 (3.89)	14 (5.6)
Marital state	Not married/not partnered	397 (11.88)	127 (18.30)	74 (29.60)
	Married/partnered	2,944 (88.12)	567 (81.70)	176 (70.40)
Social contact	No one	79 (2.36)	28 (4.03)	16 (6.4)
	Rarely meet	7 (0.21)	1 (0.14)	0 (0)
	1–6/year	218 (6.52)	87 (12.54)	22 (8.8)
	1–2/months	834 (24.96)	108 (15.56)	37 (14.8)
	1/week	812 (24.3)	142 (20.46)	59 (23.6)
	2–3/weeks	454 (13.59)	102 (14.7)	32 (12.8)
	Everyday	937 (28.05)	226 (32.56)	84 (33.6)
Personal allowance (USD)		173.39 ± 162.57	126.08 ± 154.88	107.31 ± 83.25
Depression		1.38 ± 1.45	1.61 ± 1.82	1.5 ± 1.92
MMSE score		28.15 ± 1.85	27.07 ± 2.00	27.37 ± 2.08
Smoking	No smoking	2,239 (67.02)	476 (68.59)	166 (66.4)
	Past smoking	474 (14.19)	104 (14.99)	33 (13.2)
	Current smoking	628 (18.8)	114 (16.43)	51 (20.4)
Alcohol assumption	No drinking	1,590 (47.59)	365 (52.59)	137 (54.8)
	Past drinking	326 (9.76)	80 (11.53)	28 (11.2)
	Current drinking	1,425 (42.65)	249 (35.88)	85 (34)
Exercise	Adequate (More than 150 min/week)	653 (19.55)	125 (18.01)	69 (27.60)
	Deficient (<150 min/week)	686 (20.53)	105 (15.13)	28 (11.20)
	No exercise	2,002 (59.92)	464 (66.86)	153 (61.20)
ADL		0.01 ± 0.15	0.02 ± 0.28	0.02 ± 0.20
IADL		0.13 ± 0.61	0.13 ± 0.76	0.18 ± 0.83

USD = 1,118.30 Korean Won, average exchange rate between 2010 and 2018.

MCI, mild cognitive impairment; MMSE, mini mental state examination; ADL, activity of daily living; IADL, instrumental activity of daily living.

Regarding social contact, 79 (2.36%), 28 (4.03%), and 16 (6.4%) in the normal cognition, MCI, and dementia groups, respectively, stated that they have no close social contacts. The percentage of individuals who have social contacts whom they regularly meet from once a year to 2–3 times a week was higher in the normal cognition group than in the dementia and MCI groups. However, the percentage of individuals who meet a social contact every day was the highest in the dementia group, with 84 (33.6%), 226 (32.56%), and 937 (28.05%) in the dementia, MCI, and normal cognition groups, respectively.

The number of individuals with college education or higher was 445 (13.32%), 27 (3.89%), and 14 (5.6%) in the normal cognition, MCI, and dementia groups, respectively. Regarding marital status, the number of people living alone due to bereavement or divorce was 74 (29.60%), 127 (18.30%), and 397 (11.88%) in the dementia, MCI, and normal cognition groups, respectively.

Personal allowance was the highest in the normal cognition group, with 173.39 ± 162.57 USD, 126.08 ± 154.88 USD, and 107.31 ± 83.25 USD in the normal cognition, MCI, and dementia groups, respectively. Depression score was 1.38 ± 1.45, 1.61 ± 1.82, and 1.5 ± 1.92 in the normal cognition, MCI, and dementia groups, respectively. As the participants with normal cognitive function in the 3rd survey was selected, the cognitive function scores in the 3rd survey did not significantly vary among the normal cognition g (28.15 ± 1.85), MCI (27.07 ± 2.00), and dementia (27.37 ± 2.08) groups.

### Exercise status of middle-aged and older adults by cognitive function

Table 2 shows the results of a longitudinal analysis of the exercise status of the cognitive function group based on the 2018 assessment. The within percent of the entire study sample was 41.80%; that is, the individuals who claimed to engage in adequate exercise maintained an adequate level of exercise for ~41.80% (3 years) of the 8-year period. By cognitive function group, the normal cognition group maintained an adequate level of exercise for 42.57% of the 8-year period, while the percentages were shorter at 39.29 and 39.8% in the MCI and dementia groups, respectively. The dementia group did not exercise at all for 76.86% of the period, which is relatively longer than that of the MCI (72.34%) and normal cognition (68.53%) groups.

**Table 2:** Exercise status of middle-aged and older adults stratified according to their cognitive function.

**Category**		**Overall *n* (%)**	**Between *n* (%)**	**Within** ***n* (%)**
Total	1	13,291 (62.0)	3,811 (88.9)	69.8
	2	3,827 (17.9)	2,044 (47.7)	37.5
	3	4,307 (20.1)	2,061 (48.1)	41.8
Normal (*N* = 3,341)	1	9,591 (59.7)	2,799 (87.2)	68.5
	2	3,129 (19.5)	1,626 (50.6)	38.5
	3	3,335 (20.8)	1,567 (48.8)	42.6
MCI (*N* = 694)	1	2,286 (67.9)	632 (93.9)	72.3
	2	466 (13.9)	279 (41.5)	33.4
	3	613 (18.2)	312 (46.4)	39.3
Dementia (*N* = 250)	1	857 (74.5)	223 (97.0)	76.9
	2	92 (8.0)	65 (28.3)	28.3
	3	201 (17.5)	101 (43.9)	39.8

**Figure 1:**
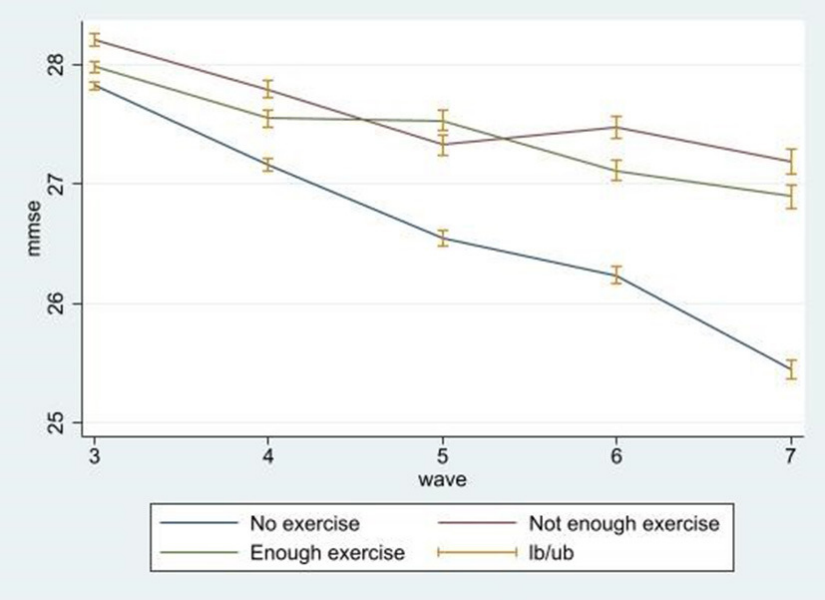
Longitudinal trends of cognitive function by exercise group.

Figure 1 illustrates the changes in cognitive function scores over the 8-year period from the 3rd wave to the 7th wave. The green line is the cognitive function score of the adequate exercise group, and the red line represents the cognitive function score of the deficient exercise group. The blue line represents the cognitive function score of the no exercise group. The cognitive function score tended to decline in all groups, and although the graph does not present causality, the cognitive function score decreases more dramatically in the no exercise group, as compared to the other two groups

### Fixed effects model for cognitive score according to WHO-defined adequate exercise

Table 3 shows the results of a fixed effects model analysis of the effects of an adequate exercise level according to the WHO definition on the changes in cognitive function in the entire study sample. We investigated the effects of each factor identified in previous studies to affect cognitive function, namely demographic characteristics, personal allowance, depression, and health behaviors. This model was statistically significant (*F* = 108.15, *p* < 0.001).

**Table 3:** Effects of exercise on cognition by a fixed effects linear regression analysis of panel data obtained from 2010 to 2018 KLoSA.

**Independent variables**		**Dependent variable: MMSE score**					
		***B***	**SE**	***t***	***p***	**[95% confidence interval]**	
Age		−0.112	0.004	−30.250	<0.001***	−0.120	−0.105
Sex (reference: male)		–	–	–	–	–	–
Education		0.438	0.035	12.440	<0.001***	0.369	0.507
Marital state (reference: not married)		0.312	0.081	3.840	<0.001***	0.152	0.471
Social contact		0.116	0.015	7.990	<0.001***	0.087	0.144
Personal allowance		0.002	0.002	1.000	0.316	−0.002	0.005
Depression		−0.176	0.013	−13.110	<0.001***	−0.203	−0.150
Smoking		0.030	0.053	0.560	0.578	−0.075	0.134
Alcohol assumption		0.034	0.039	0.880	0.381	−0.043	0.111
Exercise sufficiency (reference: no exercise)	Not enough	0.289	0.057	5.040	<0.001***	0.177	0.402
	Enough	0.366	0.056	6.550	<0.001***	0.256	0.475
ADL		−0.549	0.061	−8.940	<0.001***	−0.672	−0.425
IADL		−0.793	0.026	−30.120	<0.001***	−0.845	−0.742
Wave (reference: 3rd wave)	4	−0.376	0.055	−6.790	<0.001***	−0.485	−0.268
	5	−0.509	0.058	−8.800	<0.001***	−0.623	−0.396
	6	−0.540	0.061	−8.870	<0.001***	−0.660	−0.421
	7	−0.892	0.065	−13.680	<0.001***	−1.020	−0.764
Constant		33.158	0.367	90.430	<0.001***	32.439	33.876

Sex is omitted due to collinearity.

***p <0.001, **p <0.01, and *p <0.05.

A fixed effects model was used to estimate the effects of changes of an independent variable on the changes of the dependent variable. As known thus far, old age is the most powerful predictor of cognitive decline, and low educational level, female sex, unmarried status compared to married status or living with a partner, and low social contact frequency were identified as statistically significant predictors of cognitive decline (*p* < 0.001). Contrarily, smoking and alcohol-related factors were not significant predictive factors (*p* > 0.05). In addition, increasing depression and strong dependence when performing ADL and IADL were found to have adverse effects on cognitive decline (*p* < 0.001). Personal allowance was not significantly associated with cognitive changes.

Regarding exercise adequacy, the primary outcome of this study, deficient exercise had statistically significant effects on the cognitive function score (*B* = 0.289, *p* < 0.001); similar results were observed for adequate exercise with no exercise as the reference. The coefficient size was larger with adequate exercise than with deficient exercise (*B* = 0.366, *p* < 0.001).

Table 4 shows the fixed effects estimations for each of the cognitive function groups as assessed in the final 7th survey. As we used a fixed effects model, and sex is an invariant factor, the regression coefficient was not computed. Educational level had significant effects on the changes in cognitive function among those assessed to have a normal cognitive function in the last assessment but did not have significant effects in the dementia or MCI group. Age had significant negative effects on cognitive function in all groups. Marital status can be changed over the 8-year period, and the married status had positive effects on cognitive function compared to the unmarried status in the dementia group but not in the MCI and normal cognition groups. Social contact frequency did not have significant effects on the longitudinal cognitive decline in the normal cognition group (although this group had the highest social contact frequency) but had statistically significantly positive effects in the MCI and dementia groups, although the coefficients were not large. Smoking and alcohol consumption did not have statistically significant effects on longitudinal cognitive decline over the 8-year period. Depression had statistically significant effects in all groups, with the dementia group having the largest coefficient in the final assessment. One interesting finding was that ADL was not significantly associated with cognitive changes in the dementia and MCI groups. Increased dependence for ADL was linked to cognitive decline in the normal cognition group. However, increasing dependence for IADL was associated with cognitive decline in all groups. Increased personal allowance was not significantly associated with cognitive decline over time.

**Table 4:** Effects of exercise on cognition using fixed effects linear regression by cognitive group.

**MMSE**	**Normal**						**MCI**						**Dementia**					
	**Coefficient**	**Std. err**.	***t***	***P* > |*t*|**	**[95% conf. interval]**		**Coefficient**	**Std. err**.	***t***	***P* > |*t*|**	**[95% conf. interval]**		**Coefficient**	**Std. err**.	***t***	***P* > |*t*|**	**[95% conf. interval]**	
Sex	−0.039	0.019	−2.05	0.04*	−0.075	−0.002	−0.241	0.055	−4.35	<0.001***	−0.350	−0.133	−0.208	0.147	−1.42	0.155	−0.496	0.079
Education	0.378	0.028	13.27	<0.001***	0.322	0.434	0.141	0.089	1.58	0.114	−0.034	0.315	0.169	0.252	0.67	0.504	−0.326	0.663
Age	−0.032	0.003	−10.29	<0.001***	−0.038	−0.026	−0.175	0.009	−18.98	<0.001***	−0.194	−0.157	−0.188	0.023	−8.1	<0.001***	−0.233	−0.143
Marital state	−0.053	0.070	−0.76	0.447	−0.191	0.084	0.273	0.179	1.52	0.128	−0.078	0.624	1.260	0.456	2.76	0.006**	0.367	2.154
Social contact	0.015	0.013	1.2	0.229	−0.010	0.040	0.214	0.034	6.23	<0.001***	0.147	0.281	0.435	0.090	4.83	<0.001***	0.259	0.612
Smoking	0.094	0.044	2.14	0.032	0.008	0.180	−0.018	0.132	−0.14	0.890	−0.277	0.240	0.126	0.363	0.35	0.729	−0.586	0.838
Alcohol assumption	−0.002	0.032	−0.07	0.945	−0.064	0.060	−0.038	0.097	−0.39	0.698	−0.228	0.152	0.029	0.281	0.1	0.918	−0.521	0.579
Depression	−0.119	0.012	−9.58	<0.001***	−0.143	−0.095	−0.179	0.032	−5.6	<0.001***	−0.242	−0.116	−0.260	0.077	−3.36	0.001**	−0.412	−0.108
ADL	−0.562	0.076	−7.36	<0.001***	−0.712	−0.412	−0.326	0.170	−1.92	0.057	−0.663	0.010	−0.084	0.173	−0.48	0.629	−0.424	0.256
IADL	−0.172	0.031	−5.5	<0.001***	−0.234	−0.111	−0.355	0.065	−5.46	<0.001***	−0.482	−0.228	−1.227	0.085	−14.47	<0.001***	−1.393	−1.061
Personal allowance (USD)	0.000	0.001	<0.001	0.996	−0.002	0.002	−0.006	0.005	−1.17	0.240	−0.015	0.004	−0.024	0.020	−1.2	0.231	−0.063	0.015
Exercise sufficiency (reference: no exercise)	0.083	0.047	1.78	0.075	−0.008	0.175	0.183	0.173	1.06	0.290	−0.156	0.522	2.914	0.608	4.79	<0.001***	1.723	4.105
	0.048	0.047	1.02	0.307	−0.044	0.141	0.376	0.158	2.39	0.017*	0.067	0.686	2.803	0.450	6.23	<0.001***	1.921	3.685
Constant	29.325	0.300	97.89	<0.001***	28.737	29.912	36.510	0.913	39.99	<0.001***	34.721	38.299	33.958	2.369	14.33	<0.001***	29.315	38.602
*F* (*p*-value)	403.09 (<0.001***)						467.07 (<0.001***)						72.17 (<0.001***)					

***p < 0.001, **p < 0.01, *p < 0.05.

Regarding exercise adequacy, the study's primary outcome, adequate and deficient exercise had statistically significantly more positive effects on cognitive function, as compared to no exercise. However, the coefficient size was not larger in the adequate exercise group than in the deficient exercise group. In the MCI group, with no exercise as the reference, adequate exercise had significantly positive effects. However, deficient exercise did not have significant effects. In the normal cognition group, exercise adequacy did not have significant effects on cognitive changes.

## Discussion

This study longitudinally analyzed the levels of exercise among middle-aged and older adults with normal cognition, MCI, or dementia in Korea from 2010 to 2018 and investigated the effects of an adequate exercise level on cognitive function using a fixed effects model. The neuropathological complexity of dementia is a barrier to dementia prevention, and the effects of lifestyle modification in delaying cognitive decline remain unclear with inconsistent findings. This study broadly has three implications: 1) for adults with a gene specific for dementia onset, continuous exercise helps in slowing the cognitive decline over time, 2) exercise has significant positive effects on cognitive function in the MCI and dementia groups; however, there is low evidence on the effectiveness of exercise in older adults with normal cognition, and 3) according to the WHO guidelines, adequate exercise and deficient exercise exert different effects in preventing cognitive decline in each of the cognitive function groups.

First, the results of the fixed effects model used in this study are significant, in that they control for the unique individual characteristics that crucially contribute to cognitive decline and dementia onset, such as genetic factors (related to beta-amyloid accumulation). On the basis of our findings, even those with a gene specific for dementia onset, continuous exercise helps slow the cognitive decline over time, as compared to no exercise at all. Therefore, we can infer that an adequate exercise level is a significant protective factor against cognitive decline over time even after controlling for unique individual characteristics. One supportive evidence recently published reported that patients with Alzheimer's disease did not develop dementia symptoms, supporting the fact that cognitive function has resilience (22–24). It is hypothesized that a healthy lifestyle delays the onset of dementia and its symptoms by increasing the brain's resilience. Duzel et al. (25) shed light on the existence of exercise-related neuroplasticity and showed that continuous exercise can boost brain functions, prevent hippocampal atrophy, and induce structural and neurochemical changes of the brain area involved in memory using MRI scans.

Second, the WHO currently recommends moderate exercise for older adults with normal cognition; however, it suggests that there is low evidence on the effectiveness of exercise in older adults with a cognitive decline (11). However, our findings showed that exercise did not have statistically significant effects on cognitive decline over time, regardless of the exercise duration in older adults with a normal cognitive function, whereas educational level, depression, ADL, and IADL were actually associated with cognitive decline over time. Contrarily, exercise had significantly positive effects on cognitive function in the MCI and dementia groups. The Cochrane review, which meta-analyzed the effect of aerobic exercise on cognitive function in normal elderly without cognitive impairment, showed that some individuals had delayed memory function and attention skills; however, 9 out of 11 cognitive function areas showed no statistically significant positive effects compared with the no intervention group (24, 26). In this study, the sub-domains of cognitive function could not be identified, so comparison with previous studies was not possible. The studies included in the Cochrane review were RCTs, but the follow-up period was short (8–26 weeks). In this study, 8-year follow-up of a large panel survey was used to improve such aspects, but the overall improvements in cognitive function could not be confirmed.

Further, the coefficient size was larger and significant at *p* < 0.001 in the MCI group than in the dementia group. These results highlight the need for continuous exercise intervention in older adults with MCI or dementia. Moreover, our study findings would serve as fundamental data for prospective systematic reviews and meta-analyses of studies relating to exercise interventions for older adults with MCI and dementia.

Third, this study analyzed whether adequate and deficient exercise according to the WHO guidelines differ in their effects in preventing cognitive decline in each of the cognitive function groups. We reviewed the existing studies but could not identify studies that compared adequate and deficient exercises. A recent systematic review did not also find a dose–response association due to the large heterogeneity in the frequency, duration, and type of exercise interventions (19, 27). According to our results, adequate exercise—regardless of the exercise type—served as a protective factor against MCI and dementia, as compared to no exercise at all. However, one finding that complicates our interpretation on the exercise duration is that the effects of deficient exercise (<150 min/week) did not statistically significantly differ with those of no exercise in the MCI group. The study results pertaining to exercise interventions for older adults with MCI remain controversial. A systematic review showed that 92% of the 14 included studies reported statistically non-significant effects and that exercise does not improve memory (28, 29). In a well-designed study included in a systematic review on MCI, 1-year follow-up of the moderate aerobic exercise and sedentary groups showed no significant differences in terms of cognitive functions; however, more poorly designed studies reported mixed findings (28). Based on our findings, in the dementia group, both adequate and deficient exercises were effective as compared with no exercise (Deficient exercise: *B* = 2.914, *p* < 0.001; Adequate exercise: *B* = 2.803, *p* < 0.001). As in previous studies, this finding may support the hypothesis that exercise intensity rather than exercise duration will help maintain and improve cognitive function (30, 31).

Based on the abovementioned results, exercise may have varying effects on the cognitive function among the normal cognition, MCI, and dementia groups, and appropriate exercise intensity and degree may differ among these groups. Overall, scientific evidence for the association between exercise and cognitive function is still insufficient, but our results suggest that continuous exercise has positive effects on cognitive function longitudinally, as compared to not exercising at all. As prevention is the best treatment, it is important to manage modifiable risk factors and to continuously implement exercise interventions not only in older adults with normal cognitive function but also in those with MCI and dementia (6).

The main limitations of this study are the absences of exercise type consideration and the exercise evaluative numerical measurement such as VO2max (Maximal oxygen consumption) or METs (Metabolic equivalent of task) values. In the future, longitudinal follow-up of a large population with parameters that enable the determination of exercise intensity specifically for different exercise types, such as resistance and aerobic exercises (e.g., metabolic equivalents) would facilitate the development of evidence-based guidelines for physical activities that enhance cognitive functions in older adults.

## Conclusion

This study showed that the adequacy of exercise longitudinally over an 8-year period could have positive effects on the cognitive function scores in older adults with dementia. Further, our result showing that the coefficient size of the effect was larger in the dementia and MCI groups than in the normal cognition group highlights the importance of implementing regular exercise interventions in cognitively impaired older adults. However, exercising for ≥150 min/week was not found to be more effective than that for <150 min/week; thus, we could not determine the benefits of exercise durations that meet or exceed the WHO guidelines. Since we only examined the total weekly exercise duration without considering the exercise type, we recommend that large-scale longitudinal studies that take exercise intensity and type into consideration should be conducted in the future.

## Data availability statement

Publicly available datasets were analyzed in this study. This data can be found here: Korean Longitudinal Study of Aging (KLoSA). https://survey.keis.or.kr/eng/klosa/klosa01.jsp.

## Author contributions

DK and YK contributed to conception and design of the study. DK organized the database, performed the statistical analysis, and wrote the first draft of the manuscript. DK, YK, and AJ wrote sections of the manuscript. All authors contributed to manuscript revision, read, and approved the submitted version.

## Conflict of interest

The authors declare that the research was conducted in the absence of any commercial or financial relationships that could be construed as a potential conflict of interest.

## Publisher's note

All claims expressed in this article are solely those of the authors and do not necessarily represent those of their affiliated organizations, or those of the publisher, the editors and the reviewers. Any product that may be evaluated in this article, or claim that may be made by its manufacturer, is not guaranteed or endorsed by the publisher.
